# Supplementary material for: E/Z Molecular Photoswitches Activated by Two-Photon Absorption: Comparison between Different Families
Source: Molecules. 2021 Dec 5;26(23):7379. doi: 10.3390/molecules26237379 (PMC8659108; doi:10.3390/molecules26237379)
Supplement: Supplementary file 1 [file molecules-26-07379-s001.zip › molecules-1466315-supplementary.pdf]

Supporting Information

E/*Z* Molecular Photoswitches Activated by Two-Photon Absorption: Comparison between Different Families

Marco Marazzi [1,2,*], Cristina García-Iriepa [1,2,*], Carlos Benitez-Martin [3,4], Francisco Najera [3,4], Antonio Monari [5,6] and Diego Sampedro [7,*]

1 Departamento de Química Analítica, Química Física e Ingeniería Química, Universidad de Alcalá, Ctra. Madrid-Barcelona, Km 33.600, E-28805, Alcalá de Henares, Madrid, Spain;
2 Instituto de Investigación Química "Andrés M. del Rio" (IQAR), Universidad de Alcalá, Ctra. Madrid-Barcelona, Km 33.600, E-28805, Alcalá de Henares, Madrid, Spain;
3 Universidad de Málaga-IBIMA, Departamento de Química Orgánica, 29071 Málaga Spain;
4 Centro Andaluz de Nanomedicina y Biotecnología (BIONAND), Parque Tecnológico de Andalucía, 29590 Málaga, Spain;
5 Université de Lorraine & CNRS, LPCT, F-54000 Nancy France;
6 Université de Paris and CNRS, ITODYS, F-75006 Paris, France ;
7 Departamento de Química, Centro de Investigación en Síntesis Química, Universidad de La Rioja, Madre de Dios, 53, E-26006, Logroño, Spain

* Correspondence: marco.marazzi@uah.es (M.M.); cristina.garciai@uah.es (C.G.-I.); diego.sampedro@unirioja.es (D.S.)

Table S1. Comparison of two different DFT functionals (CAM-B3LYP and M062X) in reproducing the spectrum of five proposed cores, including the excitation energy (λ (nm)) and the OPA intensity expressed as oscillator strength (f).

Compound	State	λ(nm)cam-B3LYP	f (cam-B3LYP)	λ(nm) M062X	f M062X
Schiff base-like (*E* isomer)	S1	275.10	0.8322	272.49	0.8291
	S2	256.46	0.0027	268.91	0.0023
	S3	250.31	0.0053	248.33	0.0067
	S4	211.94	0.0005	213.80	0.0003
	S5	207.91	0.1770	208.16	0.1738
	S6	199.68	0.1682	199.87	0.0001
Schiff base-like (*Z* isomer)	S1	275.84	0.5544	279.97	0.1800
	S2	260.40	0.1112	267.52	0.4797
	S3	247.20	0.0032	245.19	0.0038
	S4	212.44	0.0116	213.57	0.0099
	S5	210.31	0.0955	210.44	0.0964
	S6	205.42	0.0734	205.85	0.0423
	S7	202.83	0.0950	201.10	0.0975
Protonated Schiff base-like (*E* isomer)	S1	333.63	0.8500	334.65	0.8376
	S2	315.40	0.0204	314.12	0.0196
	S3	220.36	0.0428	219.71	0.0412
	S4	209.71	0.0097	211.65	0.0001
	S5	206.31	0.0000	209.09	0.0097
	S6	200.54	0.0100	205.05	0.0003
Protonated Schiff base-like (*Z* isomer)	S1	362.52	0.5256	363.36	0.5168
	S2	329.09	0.0217	327.24	0.0188
	S3	234.30	0.0740	234.69	0.0741
	S4	218.23	0.0084	217.12	0.0084
	S5	208.85	0.0605	212.39	0.0060
	S6	206.58	0.0062	208.37	0.0005
Oxazolone-like (*E* isomer)	S1	317.84	0.6047	315.48	0.6069
	S2	287.54	0.0002	292.99	0.0002
	S3	275.77	0.0272	272.83	0.0247
	S4	226.50	0.0456	223.77	0.0436
	S5	218.73	0.0006	217.16	0.0009
	S6	212.66	0.0952	212.70	0.1004
Oxazolone-like (*Z* isomer)	S1	312.97	0.7389	310.92	0.7368
	S2	285.90	0.0005	290.17	0.0005
	S3	271.54	0.0212	269.16	0.0204
	S4	226.20	0.0031	223.52	0.0050
	S5	218.75	0.0004	218.95	0.0006
	S6	212.12	0.0925	212.18	0.0969
Hydantoin-like (*E* isomer)	S1	314.25	0.6521	311.00	0.6518
	S2	270.77	0.0000	277.78	0.0000
	S3	264.22	0.0138	261.41	0.0112
	S4	224.59	0.0002	228.50	0.0002
	S5	217.73	0.0553	216.62	0.0854
	S6	215.50	0.1136	216.50	0.0001
Hydantoin-like (*Z* isomer)	S1	296.23	0.6753	293.89	0.6702
	S2	265.47	0.0025	272.66	0.0050
	S3	252.70	0.0030	250.69	0.0031
	S4	214.81	0.0758	217.18	0.0200
	S5	214.15	0.0014	215.34	0.0087
	S6	212.31	0.0545	213.37	0.0935
Pyrrolinone-like (*E* isomer)	S1	320.79	0.6795	319.17	0.6768
	S2	289.50	0.0040	296.12	0.0083
	S3	255.41	0.0005	254.21	0.0004
	S4	246.33	0.1233	242.44	0.1056
	S5	228.51	0.0177	230.95	0.0161
	S6	222.88	0.1001	224.14	0.0997
Pyrrolinone-like (*Z* isomer)	S1	315.49	0.7555	314.14	0.7468
	S2	287.04	0.0073	293.98	0.0145
	S3	256.23	0.0020	255.05	0.0020
	S4	246.28	0.0514	242.22	0.0490
	S5	221.85	0.0152	224.24	0.0079
	S6	220.63	0.1017	221.68	0.0972

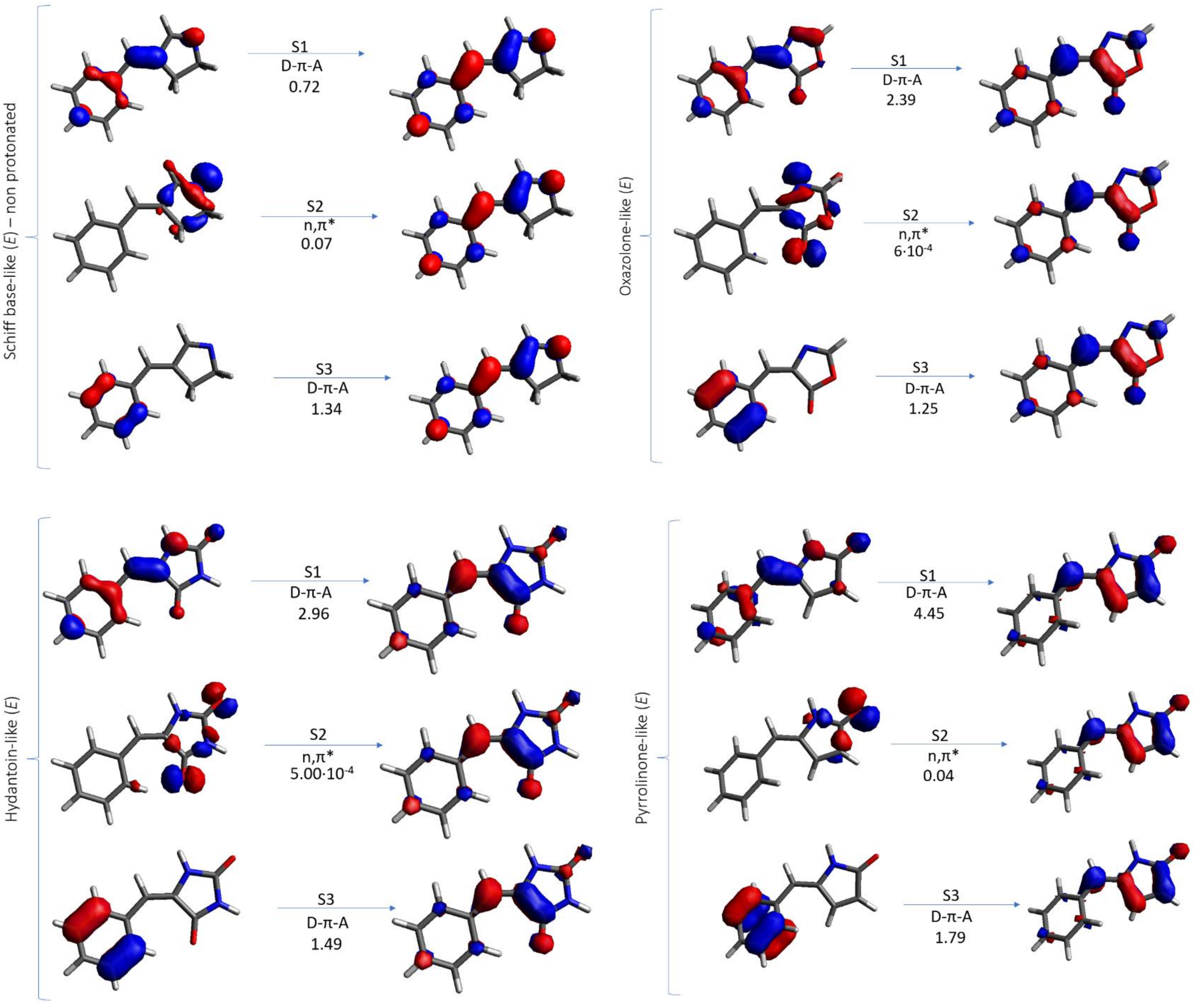

Figure S1. Molecular orbitals mainly involved in the lowest-in-energy optical transitions ($S_0 \rightarrow S_{1,2,3}$) for the *E* form of the non-protonated Schiff base-like (up left), the oxazolone-like (up right), the hydantoin-like (bottom left), and the pyrrolinone-like (bottom right) cores. The σ^{TPA} values in GM are given below each arrow.

Table S2. One-photon (OPA) and Two-photon (TPA) photophysical data of the protonated or methylated Schiff base-like compounds in air equilibrated acetonitrile solutions.

**Compound**		λ_{abs}^{OP} **(nm)** [a]	λ_{em}^{OP} **(nm)** [b]	ϕ_F [c]	σ^{TPA} **(GM)** [d] **/ [**λ_{exc}^{TP} **(nm)]**
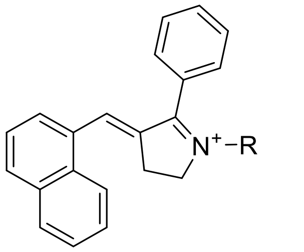	R = H	353	520	0.0221	20 / [790]<br>29 / [710]
	R = Me	350	520	0.0265	41 / [770]<br>52 / [710]
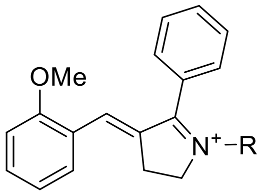	R = H	369	520	0.0067	10 / [790]<br>15 / [710]
	R = Me	370	520	0.0030	15 / [790]<br>17 / [710]
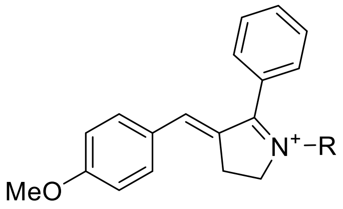	R = Me	371	510	0.0010	9 / [750]<br>14 / [710]

[a] Longest-wavelength absorption maximum. [b] Fluorescence emission maximum. [c] Fluorescence emission quantum yield (see experimental section for details). [d] TPA cross-sections measured in the range of 700-1000 nm.

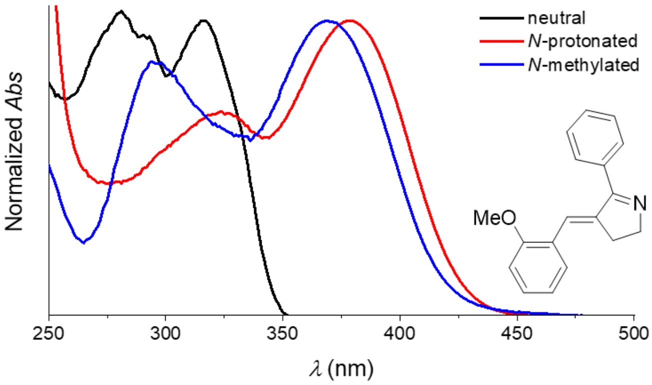

Figure S2. One-photon absorption (OPA) spectra of the Schiff base-like photoswitch (R_1: -(*o*OMe)Ph. R_2: Ph) in different forms. The other OPA spectra are already shown in ref. 70.

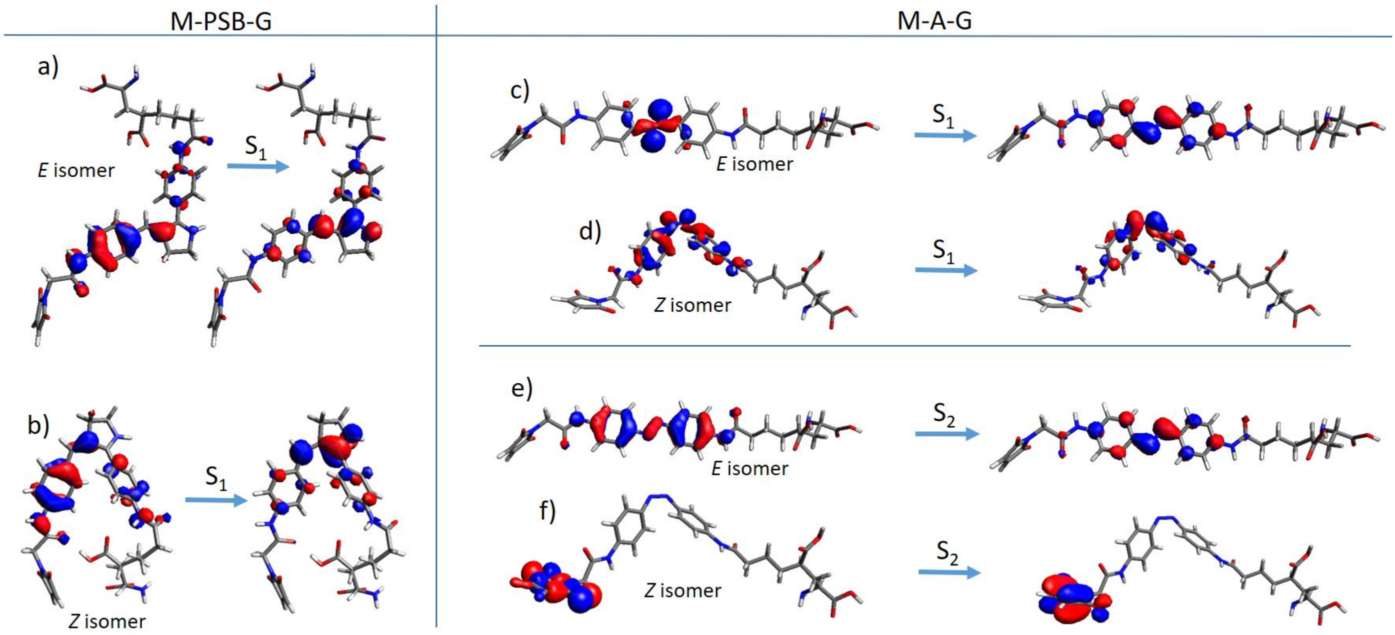

Figure S3. Molecular orbitals describing the M-PSB-G $S_0 \rightarrow S_1$ transition of a) *E* and b) *Z* isomers; molecular orbitals describing the M-A-G $S_0 \rightarrow S_1$ transition for both isomers (c, d), as well as the $S_0 \rightarrow S_2$ transition (e, f).
